# Compound‐specific ^15^N analysis of amino acids: A tool to estimate the trophic position of tropical seabirds in the South China Sea

**DOI:** 10.1002/ece3.4282

**Published:** 2018-08-11

**Authors:** Libin Wu, Xiaodong Liu, Liqiang Xu, Linjie Li, Pingqing Fu

**Affiliations:** ^1^ Institute of Polar Environment School of Earth and Space Sciences University of Science and Technology of China Hefei Anhui China; ^2^ Anhui Province Key Laboratory of Polar Environment and Global Change University of Science and Technology of China Hefei Anhui China; ^3^ School of Resources and Environmental Engineering Hefei University of Technology Hefei Anhui China; ^4^ LAPC Institute of Atmospheric Physics Chinese Academy of Sciences Beijing China; ^5^ Institute of Surface‐Earth System Science Tianjin University Tianjin China

**Keywords:** amino acids, compound‐specific isotope analysis, multi‐TDF_G__lu‐Phe_ approach, South China Sea, trophic position, tropical seabirds

## Abstract

Compound‐specific ^15^N analysis of amino acids (AAs) is a powerful tool to determine the trophic position (TP) of organisms. However, it has only been used in a few studies of avian ecology because the AA patterns in the consumer‐diet nitrogen trophic discrimination factor (TDF_G__lu‐Phe_ = ∆^15^ N_G__lu_−∆^15^ N_P__he_) were unknown in birds until recently, and tropical seabirds have never been investigated with this methodology. Here, we explore the application of this method to tropical seabirds. In this study, we recovered the fossilized bones of tropical seabirds from ornithogenic sediments on two coral islands in the Xisha Islands, South China Sea, as well as the bones and muscle of their predominant food source, flying fish (*Exocoetus volitans*). Compound‐specific ^15^N and ^13^C analyses of AAs in both seabird and fish bone collagen were conducted. The TP of flying fish was calculated based on a widely used single TDF_G__lu‐Phe_ approach. We then calculated the TP of tropical seabirds in three different ways: (a) according to the composition of their diet; (b) based on the single TDF_G__lu‐Phe_ approach; and (c) using a multi‐TDF_G__lu‐Phe_ approach. The results of the multi‐TDF_G__lu‐Phe_ approach were much closer to the results based on the composition of the seabird diet than the results of the single TDF_G__lu‐Phe_ approach, confirming its applicability for tropical seabirds. For seabird bone samples of different ages, TP determined from the multi‐TDF_G__lu‐Phe_ approach was most similar to that of bulk δ^15^N of bird collagen, with seabirds occupying higher TPs during the Little Ice Age, as previously shown. In addition, the ^13^C Suess effect was reflected in the AAs δ^13^C in our samples. This study applied a compound‐specific ^15^N analysis of AAs to determine the TP of tropical seabirds that has potential to extend to all tropical seabirds many of which are widely distributed and play a key role in the evolution of coral island ecosystems.

## 1 INTRODUCTION

Stable isotope analysis is a widely used technique in ecology (Fry, 2006), especially for investigations of long‐ranging species. It has been demonstrated that δ^15^N is a robust marker of trophic position (TP) due to its general increase in organisms along the food chain, when nitrogen fractionates from prey to predator (Hobson, 1999; Post, 2002). With an increase in one trophic level, the average δ^15^N in tissues tends to be enhanced by about 3.4‰ (DeNiro & Epstein, 1981; Minagawa & Wada, 1984; Post, 2002). Therefore, we can estimate the TP of a consumer by a simple formula: TP_consumer_ = (δ^15^N_consumer_–δ^15^N_producer_)/3.4 + 1, if the organism bulk δ^15^N of both the consumer (δ^15^N_consumer_) and producer (δ^15^N_producer_) are known. However, δ^15^N_producer_ is sometimes difficult to determine because there are temporal and spatial variations in the isotope baseline (producers) and adequate sampling is essential. Thus, we cannot compare the TPs of organisms from different regions if only their δ^15^N values are known, and a correction based on δ^15^N_producer_ or background δ^15^N is also needed (Navarro, Coll, Somes, & Olson, 2013). Moreover, because there are likely to be various δ^15^N values in different tissues of the same organism (e.g., Cano‐Rocabayera, Maceda‐Veiga, & de Sostoa, 2015; Hobson, 1995), determining the actual TP becomes more complex.

To solve these problems, a compound‐specific ^15^N analysis of amino acids (AAs) has been applied to estimate the TP of organisms (Chikaraishi et al., 2009; McCarthy, Benner, Lee, & Fogel, 2007; McClelland & Montoya, 2002; Ohkouchi et al., 2017). AAs can be divided into two types according to whether the carbon–nitrogen bond cleaves during metabolic transamination accompanied by an obvious isotopic fractionation. Some AAs, including alanine (Ala), valine (Val), isoleucine (Ile), and glutamic acid (Glu), are called “trophic” AAs because they are enriched in δ^15^N (as high as 10‰) when carbon–nitrogen bonds cleave, whereas other AAs are called “source” AAs, including methionine (Met) and phenylalanine (Phe), and show little change in their δ^15^N up the food chain because their dominant metabolic processes neither form nor cleave the bonds related to the nitrogen atom (Chikaraishi et al., 2009). Different formulas can be used to estimate the TP of specific groups of organisms, that is, TP_Glu/Phe_ = (δ^15^N_Glu_–δ^15^N_Phe_–β)/TDF_Glu‐Phe_ + 1, where TDF_Glu‐Phe_ is the trophic discrimination factor and assumed to be 7.6 commonly, β represents the difference in δ^15^N between the Glu and Phe of primary producers, and differs for C_3_, C_4_, and aquatic food webs (β = 3.4 for aquatic food webs) (Chikaraishi, Ogawa, & Ohkouchi, 2010; Chikaraishi et al., 2009). The TP_Glu/Phe_ is based on the δ^15^N values in Glu and Phe (δ^15^N_Glu_ and δ^15^N_Phe_). This method has been widely used to estimate the TPs of aquatic organisms (Bradley et al., 2015; Chikaraishi, Kashiyama, Ogawa, Kitazato, & Ohkouchi, 2007; Chikaraishi, Steffan, Takano, & Ohkouchi, 2015; Chikaraishi et al., 2014; Nielsen, Popp, & Winder, 2015; Ohkouchi, Tsuda, Chikaraishi, & Tanabe, 2013; Zhang et al., 2016), herbivorous mammals (Ishikawa, Hayashi, Sasaki, Chikaraishi, & Ohkouchi, 2017; Itahashi, Chikaraishi, Ohkouchi, & Yoneda, 2014; Schwartz‐Narbonne, Longstaffe, Metcalfe, & Zazula, 2015; Styring, Sealy, & Evershed, 2010), and humans (Naito et al., 2016; Styring et al., 2010) in both modern‐day and ancient samples. Additionally, δ^15^N values in proline (Pro) and Phe have been used to estimate the TP of some consumers, for example, Weddell seal (Hückstädt, McCarthy, Koch, & Costa, 2017). However, these formulas are applicable to avian studies only after improving the values of TDF_Glu‐Phe_ and β. An improved formula is necessary and a multi‐TDF calculation is required for such studies because of variations in the TDF between animals within a food web (Germain, Koch, Harvey, & McCarthy, 2013; Hoen et al., 2014).

Using AAs isolated from penguin chick blood, Lorrain et al. (2009) found that the δ^15^N values of AAs can be used to estimate the relative trophic levels of penguins, but the trophic enrichment factors (TEFs) reported in previous studies were not appropriate to calculate the absolute trophic level for seabirds. McMahon, Polito, Abel, McCarthy, and Thorrold (2015) calculated an avian‐specific nitrogen TDF_Glu‐Phe_ of 3.5 ± 0.4‰, which was significantly lower than the previously reported literature TDF_Glu‐Phe_ value of 7.6‰ (Chikaraishi et al., 2007) after conducting a controlled compound‐specific stable isotope analysis in a feeding experiment on the Gentoo penguin (*Pygoscelis papua*), and examining the patterns in individual AA stable isotope fractionations between diet and consumer. This could be because of the minimal fractionation of the source AA (Phe), which always has low δ^15^N values along the chain, and relatively low trophic fractionation of the trophic AA (Glu). A new formula was then proposed to calculate the TP of birds, that is, TP_CSIA‐multi TDF_ = 2+ (δ^15^N_Glu_–δ^15^N_Phe_–TDF_(Glu‐Phe) plankton_–β)/TDF_(Glu‐Phe) penguin_, where TDF_(Glu‐Phe) plankton_ = 7.6‰, TDF_(Glu‐Phe) penguin_ = 3.5‰, and β = 3.4‰ (McMahon et al., 2015). Recently, Hebert et al. (2016) also found that the TDFs based upon the source (Phe) and trophic (Glu) AAs were 4.1 and 5.4 for muscle and red blood cells, respectively, from laboratory and field studies of captive American kestrels and wild herring gulls. This was lower than the values reported for metazoan invertebrates. Besides, stable isotope analyses of feather AAs have also been used to identify penguin migration strategies (Polito et al., 2017). There have only been a few other studies that have focused on avian species through compound specific ^15^N analyses of AAs (e.g., Quillfeldt et al., 2017).

In addition to δ^15^N, stable carbon isotopes (δ^13^C) have been used in many ecological studies to both confirm and further refine the broad interpretations made using bulk isotopic data (e.g., Webb et al., 2018). Generally, AAs are either essential or nonessential. Animals must acquire essential AAs directly from their food (Eagle, 1959). In previous studies, the δ^13^C values of essential AAs usually showed little or no trophic enrichment between predators and their food, and the TEFs for δ^13^C values of nonessential AAs were likely to be relevant to the composition and quality of the diet (Howland et al., 2003; McMahon, Fogel, Elsdon, & Thorrold, 2010; McMahon et al., 2015). Thus, δ^13^C values in AAs, especially essential AAs, have the potential to infer habitat use and source organism production (e.g., Arthur, Kelez, Larsen, Choy, & Popp, 2014; Paolini, Ziller, Laursen, Husted, & Camin, 2015) just like bulk δ^13^C values (DeNiro & Epstein, 1978). However, many studies have shown that δ^13^C values in inorganic and organic materials have decreased rapidly since 1850 AD as a result of the ^13^C Suess effect, which is caused by fossil fuel combustion and the emission of carbon with fewer ^13^C isotopes (Blight, Hobson, Kyser, & Arcese, 2015; Pereira et al., 2018), for example, δ^13^C decreased about 1.8‰ until 2014 in the South China Sea (Jia et al., 2013; Wu, Liu, Fu, Xu, Wang, et al., 2017). It is therefore important to pay careful attention to this effect in paleo‐ecological studies using δ^13^C data (including AAs data). The application of AA δ^13^C analyses is currently expanding, but further studies are still required to determine the suitability of AA δ^13^C and δ^15^N for tracing an animal diets and estimating TP, because carbon and nitrogen isotope ratios vary among different AAs (Nielsen, Clare, Hayden, Brett, & Kratina, 2017).

Tropical seabirds play a key role in the evolution of coral island ecosystems in the tropics (Allaway & Ashford, 1984; Xu et al., 2011), but their TPs have not been analyzed using a compound‐specific stable isotope analysis of their AAs, which has a better precision and possibly can yield new ecological information. Although bulk isotopes of seabirds were analyzed (Wu, Liu, Fu, Xu, Li, et al., 2017), they could not quantify historical seabird TP without data on the δ^15^N in producers. Tropical seabirds have a simple food source in that they predominantly feed on flying fish (e.g., *Exocoetus volitans* in this study) and squid (e.g., *Loligo chinensis*; Cherel et al., 2008; Xu, Liu, & Jiang, 2014). In this study, we focused on the Xisha Islands, South China Sea, where there is an abundance of tropical seabirds (Cao, Pan, & Liu, 2007). In our previous studies, we collected a number of fossilized tropical seabird bones from this location and quantitatively calculated the composition of their diet based on a nitrogen isotope mass balance (Wu, Liu, Fu, Xu, Li, et al., 2017). The characteristics and factors influencing bulk δ^13^C and δ^15^N in the muscle and scales of the tropical seabird predominant food source (i.e., flying fish) have previously been analyzed in detail (Wu, Xu, et al., 2017). The average bulk δ^13^C of plant tissues from the Xisha Islands has also been reported (Wu, Liu, & Xu, 2017), and there is the potential for further studies to assess the possibility of seabirds feeding on plants. In this study, a hypothesis was proposed that flying fish have been the predominant food item for tropical seabirds in the Xisha Islands during the past 1,200 years, as is currently the case (Cao, 2005). We conducted a compound‐specific ^15^N and ^13^C analysis of AAs in seabird and fish bone samples to test whether the multi‐TDF_Glu‐Phe_ approach of McMahon et al. (2015) was applicable to tropical seabirds, because both tropical seabirds and penguins are marine foragers. We also investigated the potential ecological significance of AAs δ^13^C and δ^15^N at the same time, including what nitrogen and carbon TEFs in AAs indicated and how the TPs of seabirds changed in the past. For comparison, the TP of seabirds based on the composition of their diet (Wu, Liu, Fu, Xu, Li, et al., 2017) was calculated after the TP of flying fish and squid were determined, and the TP of seabirds was therefore based on a single TDF_Glu‐Phe_ approach. Our study was the first to apply a stable isotope analysis of individual AAs to tropical seabirds and could help to generalize previous studies of penguins (McMahon et al., 2015) to other seabirds worldwide.

## 2 MATERIALS AND METHODS

### 2.1 Study area and sample collection

**Figure 1:**
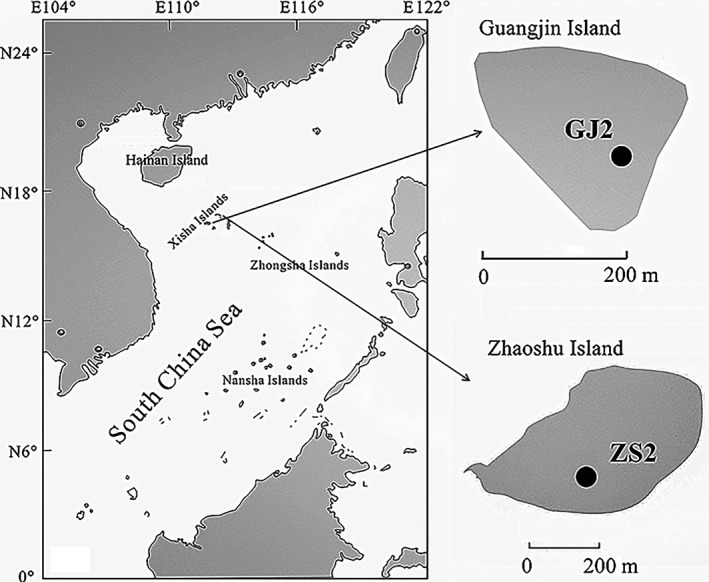
Map of the South China Sea showing sampling locations GJ2 and ZS2 at Guangjin and Zhaoshu islands, respectively

The South China Sea (3°00′ – 23°37′N, 99°10′ – 122°10′E) (Figure 1), located in the tropics, is one of the largest marginal seas in the world and is connected to the Pacific Ocean through the Luzon Strait between the Taiwan and Luzon Islands. The Xisha Islands, in the northwest South China Sea, comprise a group of about 30 islands, most of which are coral and have a typical tropical marine climate with a year‐round high temperature (the annual average temperature usually ranges from 26–27°C). The central area of some islands is covered by trees *Pisonia grandis* and *Guettarda speciose* and shrubs (*Scaevola taccada*). According to previous reports (Cao, 2005), many tropical seabirds occur on the islands, with the red‐footed booby (*Sula sula*) being the most important. Tens of thousands of red‐footed boobies inhabit Dongdao Island in the Xisha Islands (Exploration Group of Xisha Islands of Institute of Soil Science of Chinese Academy of Sciences (CAS), 1977; Hainan Ocean Administration, 1999; Cao et al., 2007).

Guangjin Island (16°27′N, 111°42′E) has an area of about 0.06 km^2^, its interior is mainly covered by *G. speciosa* and *P. grandis*, and it is bordered by the shrubs *S. taccada*,* Messerschmidia argentea*, and *Morinda citrifolia*. Zhaoshu Island (16°59′N, 112°16′E) has an area of about 0.20 km^2^; its center is mainly covered by dense patches of *S. taccada* and a small number of herbaceous *Lepturus repens* plants that grow at the margins. Unfortunately, we did not observe any seabirds on Guangjin or Zhaoshu Islands during field trips. However, a large number of guano pellets, eggshells, bird bones, fish scales, and fish bones were observed in the coral sand ornithogenic sediments underneath the dense vegetation, providing strong evidence of past seabird activity.

Sample sediment profiles, GJ2 and ZS2, were taken from Guangjin and Zhaoshu islands, respectively (Figure 1). To obtain sufficient seabird remains for analyses, a coarse fraction of sediment samples from an adjacent duplicate pit (about 1 × 1 m) was separated at intervals of 1–2 cm using a 10‐mesh stainless steel sieve *in situ*. Tropical seabird bones were sorted from these ornithogenic sediment samples and were most likely from red‐footed boobies, which is currently the most abundant species in the Xisha Islands (Cao, 2005; Wu, Liu, Fu, Xu, Li, et al., 2017). Both ^210^Pb and radiocarbon (AMS^14^C) dating were used to establish the chronology of the profiles and seabird bones. The results were reported in our earlier studies (Wu, Liu, Fu, Xu, Li, et al., 2017; Xu et al., 2016). Wu, Liu, Fu, Xu, Li, et al. (2017) suggested that these bone samples were well preserved based on their collagen C/N ratios as the ratios were within the range 2.9−3.6 (DeNiro, 1985). The composition of the diet of these tropical seabirds was determined based on a bulk stable nitrogen isotope analysis. To estimate the TP of food sources, three flying fish samples were collected around Yongxing Island (16°50′N, 112°20′E), which is close to both Guangjin and Zhaoshu islands. The samples were frozen at −20°C before defrosting and dissecting.

### 2.2 Sample preparation and analysis

Before pretreatment and stable isotope analyses, the fish were weighed and their standard length was measured. Bird and fish bones were pretreated, and their collagen was extracted using methods reported in previous studies (Brown, Nelson, Vogel, & Southon, 1988; Longin, 1971; Xu et al., 2014). The bones were cleaned using an ultrasonic bath. After cleaning, the dried bones were gently crushed into small fragments. The chemically cleaned samples were then reacted under vacuum with 1 N HCl to dissolve the bone mineral and release carbon dioxide from bioapatite. The residue was filtered, rinsed with deionized water, and heated at 80°C for 6 hr under slightly acid conditions (pH = 3) to dissolve collagen and leave humic substances in the precipitate. The collagen solution was then collected through centrifugation and dried to isolate pure collagen. Fish muscle samples were treated with (1:1) chloroform/methanol for more than 12 hr to extract and remove lipids (Inamura, Zhang, & Minagawa, 2012).

We used isotope ratio mass spectrometry (IRMS MAT 253; Thermo Fisher Scientific, Waltham, MA, USA) to analyze δ^15^N and δ^13^C levels in fish muscle samples after removing lipids (Wu, Liu, Fu, Xu, Li, et al., 2017). Collagen bulk sample δ^15^N and δ^13^C were measured using a PDZ Europa ANCA‐GSL elemental analyzer interfaced to a PDZ Europa 20‐20 IRMS (Sercon, Cheshire, UK) at the University of California, Davis (Davis, CA, USA). The stable isotopic composition of the samples was expressed in δ notation as the deviation from standards in parts per thousand (‰), δ^15^N = [(*R*_sample_/*R*_standard_) – 1] × 1000 (where *R* is the ratio ^15^N/^14^N and the *R*_standard_ value is based on atmospheric air nitrogen), and δ^13^C = [(*R*_sample_/*R*_standard_) – 1] × 1000 (‰) [where R is the ratio ^13^C/^12^C and the *R*_standard_ value is based on Vienna Pee Dee Belemnite (V‐PDB)]. Analytical precision (the standard deviation) for δ^13^C and δ^15^N was less than ±0.1‰ and ±0.2‰, respectively.

Bird and fish bone collagen extracts were also sent to UC Davis for compound‐specific ^15^N and ^13^C analysis of AAs, using the method of Walsh, He, and Yarnes (2014). Sample preparation involved acid hydrolysis for the liberation of AAs from proteins and derivatization by methyl chloroformate to produce compounds amenable to gas chromatography (GC) analyses. A condition of pH < 1 is strictly controlled by re‐suspending the dried hydrolysates in 0.4 M HCl prior to derivatization, and this had been proven to avoid uncertainty in the analysis especially for Glu (Sacks & Brenna, 2005; Yarnes & Herszage, 2017). AA derivatives were injected in splitless mode and separated on an Agilent J&W factor FOUR VF‐23 ms column (30 m × 0.25 mm ID, 0.25 μm film thickness; Agilent Technologies, Santa Clara, CA, USA). After separation, AA derivatives were finally converted to N_2_ and CO_2_ to enter the spectrometer. The final δ‐values were obtained after adjusting the provisional values to account for changes in linearity and instrumental drift, enabling the correct δ‐values for laboratory standards to be obtained. To ensure the accuracy of data, two mixtures composed of pure amino acids of calibrated δ^13^C and δ^15^N and natural materials were used as quality assurance materials and co‐measured with samples during the AAs isotopes analyses. The δ^15^N and δ^13^C of 11 AAs [Ala, aspartic acid (Asp), Glu, glycine (Gly), Ile, leucine (Leu), lysine (Lys), Met, Phe, Pro, and Val] were determined by this method.

### 2.3 Data analysis

The TP of tropical seabirds was estimated using three methods based on: (a) the composition of their diet; (b) a single TDF_Glu‐Phe_ (nitrogen isotope trophic discrimination factor between Glu and Phe) approach; and (c) a multi TDF_Glu‐Phe_ approach.

(a) First, because tropical seabirds in the Xisha Islands predominantly prey on flying fish and squid (Cao, 2005; Wu, Liu, Fu, Xu, Li, et al., 2017), the TP of tropical seabirds was inferred from their diet (TP_diet_) as follows:

(1) $${\text{TP}}_{\mathrm{diet}}=f\times {\text{TP}}_{\mathrm{flying}\;\mathrm{fish}}+\left(1-f\right)\times {\text{TP}}_{\mathrm{squid}}+1$$

where *f* represents the mass proportion of flying fish in the diet of seabirds, which was quantified in our earlier study (Wu, Liu, Fu, Xu, Li, et al., 2017). The flying fish consumed by tropical seabirds had an average muscle bulk δ^15^N value of 9.2‰ (Wu, Liu, Fu, Xu, Li, et al., 2017). The three flying fish samples had a different mass and standard length from the average values for flying fish in seabird prey (Wu, Liu, Fu, Xu, Li, et al., 2017), and there was a possible difference in the bulk muscle δ^15^N values because the size of an organism can influence tissue δ^15^N values (Olsson, Valters, & Burreau, 2000; Wu, Xu, et al., 2017). Thus, a correction was necessary and the average TP in flying fish consumed by seabirds was determined by (TP_flying fish sample_ + (9.2 − δ^15^N_flying fish sample_)/3.4), where TP_flying fish sample_, and δ^15^N_flying fish sample_ represents the average TP and muscle bulk δ^15^N values in the three flying fish samples used in this study, because TP_consumer_ = (δ^15^N_consumer_ − δ^15^N_producer_)/3.4 + 1 (Post, 2002). We adopted the single TDF_Glu‐Phe_ approach (Chikaraishi et al., 2009) to calculate the TP of flying fish as follows:

(2) $${\text{TP}}_{\mathrm{CSIA}-\mathrm{single}\;\mathrm{TDF}}=1+\left[\frac{\delta^{15}N_{\mathrm{Glu}}-\delta^{15}N_{\mathrm{Phe}}-\beta }{{\text{TDF}}_{\mathrm{Glu}-\mathrm{Phe}}}\right]$$

where δ^15^N_Glu_ and δ^15^N_Phe_ represent the stable nitrogen isotope values in bone collagen Glu and Phe, respectively, β represents the difference in the δ^15^N values between the Glu and Phe of primary producers (3.4‰ for aquatic cyanobacteria and algae), and the literature value of TDF_Glu‐Phe_ was 7.6‰ (Chikaraishi et al., 2010). Thus:

(3) $${\text{TP}}_{\mathrm{Glu}/\mathrm{Phe}\;1}=1+\left(\delta^{15}N_{\mathrm{Glu}}-\delta^{15}N_{\mathrm{Phe}}-3.4\right)/7.6$$

The TP of squid was then inferred from bulk δ^15^N values in both flying fish and squid muscle (squid in seabird prey had an average muscle bulk δ^15^N value of 10.2 ± 0.4‰, Wu, Liu, Fu, Xu, Li, et al., 2017), and the nitrogen isotope discrimination factor in the food chain, that is, the bulk δ^15^N value in tissues tended to increase to about 3.4‰ with an increase in one trophic level (DeNiro & Epstein, 1981; Minagawa & Wada, 1984; Post, 2002). The TP of tropical seabirds (TP_diet_) was then calculated using formula (1).

(b) Second, we calculated the TP of tropical seabirds (TP_Glu/Phe 1_) using the single TDF_Glu‐Phe_ approach (TP_CSIA‐single TDF_) referred to above [formula (3)].

(c) Finally, we calculated the TP of tropical seabirds (TP_Glu/Phe 2_) using a multi‐TDF_Glu‐Phe_ approach (TP_CSIA‐multi TDF_), which included an avian‐specific TDF_Glu‐Phe_ value of penguins (McMahon et al., 2015) because tropical seabirds and penguins are similar in being both seabirds and marine foragers:

(4) $${\text{TP}}_{\mathrm{CSIA}-\mathrm{multi}\;\mathrm{TDF}}=2+\left[\frac{\delta^{15}N_{\mathrm{Glu}}-\delta^{15}N_{\mathrm{Phe}}-{\text{TDF}}_{(\mathrm{Glu}-\mathrm{Phe})\mathrm{plankton}}-\beta }{{\text{TDF}}_{(\mathrm{Glu}-\mathrm{Phe})\mathrm{bird}}}\right]$$

where TDF_(Glu‐Phe)plankton_ = 7.6‰, which is typical of plankton and other lower trophic level marine organisms (e.g., Chikaraishi et al., 2007, 2009), and TDF_(Glu‐Phe) bird_ represents the avian‐specific TDF_Glu‐Phe_ value of 3.5 ± 0.4‰ based on a previously reported feeding experiment (McMahon et al., 2015). The TDF_Glu‐Phe_ value was obtained from penguin feathers, but we applied it to our bone samples because in many cases avian feathers and bone collagen have similar bulk δ^15^N values (Hobson, Alisauskas, & Clark, 1993; Huang, Sun, Long, Wang, & Huang, 2013). Therefore:

(5) $${\text{TP}}_{\mathrm{Glu}/\mathrm{Phe}\;2}=2+\left(\delta^{15}N_{\mathrm{Glu}}-\delta^{15}N_{\mathrm{Phe}}-7.6-3.4\right)/3.5$$

In our previous study (Wu, Liu, Fu, Xu, Li, et al., 2017), we calculated the composition of the diet of tropical seabirds over the past 1,200 years and compared the results between different periods, including the Medieval Warm Period (MWP, 850–1200 AD) and the Little Ice Age (LIA, 1400–1850 AD). We calculated the TP of seabirds during these and other periods using formulas (1), (3), and (5).

## 3 RESULTS

### 3.1 TP of food sources for tropical seabirds

**Figure 2:**
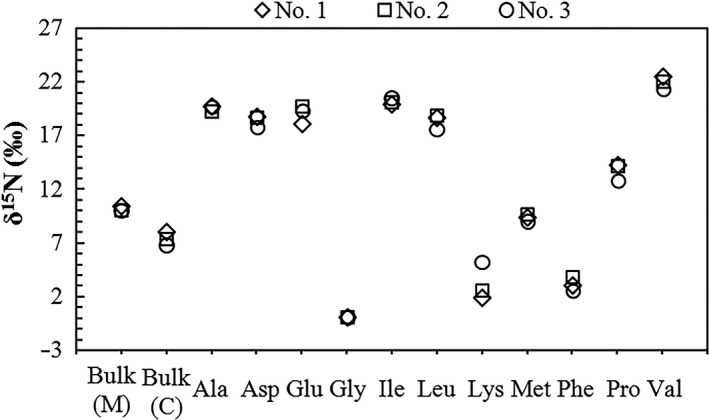
The δ^15^N values in bulk samples (muscle (M) and bone collagen (C)) and individual amino acids (AAs) (in bone collagen) of the three modern‐day flying fish samples

The results of a compound‐specific ^15^N analysis of the 11 AAs in flying fish bone collagen indicated the δ^15^N values of each AA were consistent for the three flying fish samples (Table 1, Figure 2). The TP of the fish was calculated based on the single TDF_Glu‐Phe_ approach [Formula (3)], yielding an average of 2.64 ± 0.10, with a muscle bulk δ^15^N of 10.1 ± 0.2‰. The average TP of flying fish preyed on by tropical seabirds was TP_flying fish_ = 2.38 ± 0.10 after the correction.

**Table 1:** Mass and standard length, muscle δ^15^N and δ^15^N values in bulk tissue, and individual amino acids (AAs) of bone collagen for flying fish. TP_Glu/Phe 1_ was calculated based on formula (3)

No.	1	2	3	Average
Mass (g)	334.3	300.5	241.0	291.9 ± 38.6
Standard length (cm)	29.0	28.0	25.5	27.5 ± 1.5
Muscle bulk δ^15^N (‰)	10.4	10.0	10.0	10.1 ± 0.2
Collagen bulk δ^15^N (‰)	8.0	7.3	6.7	7.3 ± 0.5
Collagen individual AAs δ^15^N (‰)				
Ala	19.7	19.2	19.7	19.6 ± 0.2
Asp	18.7	18.6	17.8	18.4 ± 0.4
Glu	18.0	19.7	19.3	19.0 ± 0.7
Gly	–0.4	–0.9	–1.8	–1.0 ± 0.6
Ile	19.9	20.1	20.4	20.2 ± 0.2
Leu	18.6	18.8	17.6	18.3 ± 0.5
Lys	1.8	2.5	5.2	3.2 ± 1.5
Met	9.3	9.6	8.9	9.3 ± 0.3
Phe	3.0	3.8	2.5	3.1 ± 0.6
Pro	14.2	14.1	12.8	13.7 ± 0.6
Val	22.5	22.0	21.3	21.9 ± 0.5
TP_Glu/Phe 1_	2.53	2.63	2.76	2.64 ± 0.10

Based on the reported average muscle bulk δ^15^N value (10.2 ± 0.4‰) of squid in seabird prey (Wu, Liu, Fu, Xu, Li, et al., 2017), the TP of squid TP_squid_ = 2.38 + (10.2–9.2)/3.4 was 2.67 ± 0.10. Because tropical seabirds predominantly prey on flying fish and squid, their TP based on the composition of their diet was TP_diet_ = 2.38 × *f* + 2.67 × (1 – *f*) + 1 = 3.67–0.29 × *f*, where *f* is the proportion of flying fish.

### 3.2 TP of ancient tropical seabirds

**Figure 3:**
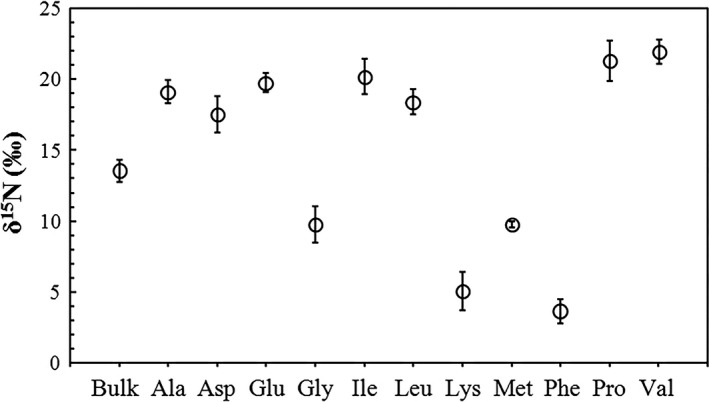
The δ^15^N values in bulk bone collagen samples and individual amino acids (AAs) from ancient tropical seabirds (*n *=* *7 individuals)

As with the flying fish samples, the overall δ^15^N values of each AA varied little in the tropical seabird bone samples (Table 2, Figure 3) and the average value of the calculated TP was 2.68 ± 0.10 and 3.44 ± 0.26 using formula (3) and (5), respectively. A statistical analysis indicated that there was a significant difference between the results from these two formulas (Student's *t*‐test, *p *<* *0.001).

**Table 2:** The δ^15^N values in bulk tissue and individual amino acids (AAs) of seabird collagen and the trophic position (TP) calculated using formulas (3) (TP_Glu/Phe 1_) and (5) (TP_Glu/Phe 2_). (“–” means no data because of an insufficient amount of sample)

No.	1	2	3	4	5	6	7	Average
Age (AD)	1913	1680	1574	1477	1341	1082	1020	
Profile	ZS2	ZS2	GJ2	GJ2	ZS2	GJ2	GJ2	
Collagen individual AAs δ^15^N (‰)								
Ala	18.8	17.8	19.6	18.7	20.3	20.1	18.4	19.1 ± 0.8
Asp	15.6	16.6	18.6	19.0	16.7	19.1	17.0	17.5 ± 1.3
Glu	19.2	19.8	20.9	19.1	19.6	20.6	19.0	19.8 ± 0.7
Gly	8.5	8.8	11.9	11.3	8.5	9.7	9.6	9.8 ± 1.3
Ile	18.6	18.1	21.0	20.8	20.1	21.7	21.0	20.2 ± 1.3
Leu	17.4	16.8	18.7	18.5	18.6	19.7	18.9	18.4 ± 0.9
Lys	3.0	7.5	6.0	5.4	4.0	5.4	4.1	5.1 ± 1.4
Met	9.7	10.1	–	–	9.5	–	–	9.8 ± 0.2
Phe	4.1	2.4	4.2	4.3	2.2	4.6	3.5	3.6 ± 0.9
Pro	19.4	19.7	23.6	21.9	21.9	22.3	20.4	21.3 ± 1.4
Val	20.9	21.1	23.7	21.7	22.2	22.0	22.0	21.9 ± 0.9
Collagen bulk δ^15^N (‰)	14.9	14.1	13.3	13.1	13.7	13.3	12.2	13.5 ± 0.8
TP_Glu/Phe 1_	2.54	2.83	2.75	2.51	2.84	2.66	2.60	2.68 ± 0.10
TP_Glu/Phe 2_	3.14	3.78	3.61	3.07	3.80	3.40	3.27	3.44 ± 0.26

### 3.3 Nitrogen and carbon trophic enrichment in AAs

**Figure 4:**
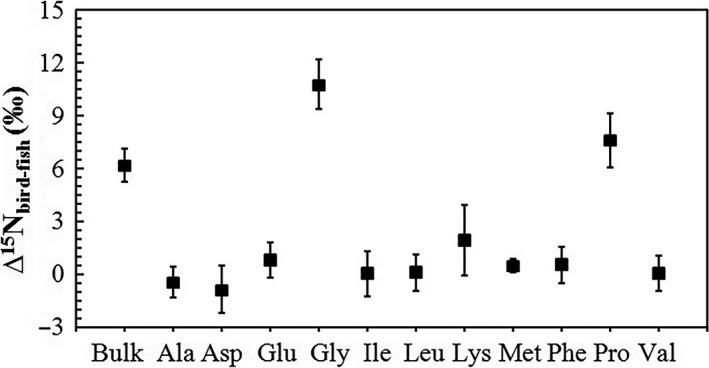
Trophic enrichment factors (TEFs) between tropical seabird and flying fish bone AAs (and bulk bone samples) δ^15^N values (*n *=* *3 for fish and *n *=* *7 for birds)

**Figure 5:**
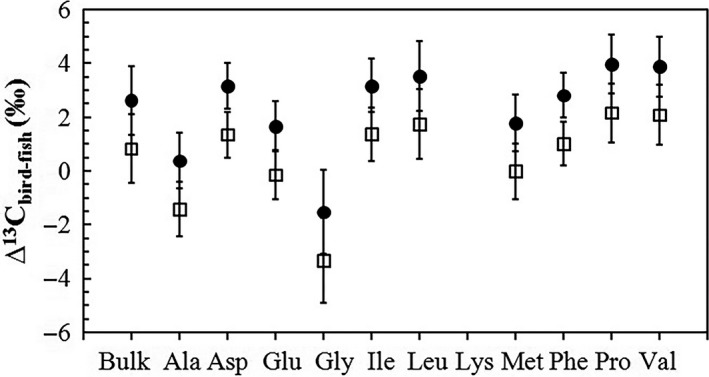
Trophic enrichment factors (TEF values) between tropical seabird and flying fish bone AAs (and bulk bone samples) δ^13^C (*n *=* *3 for fish, and *n *=* *7 for birds). The solid dots represent the results of original data, and the hollow squares are those from original data minus 1.8‰

The TEF values (∆^15^N_bird‐fish_ or ∆^13^C_bird‐fish_) for each AA or bulk sample for tropical seabird and flying fish bone δ^15^N or δ^13^C values were calculated, and the results are shown in Figures 4 and 5. For “trophic” (Ala, Asp, Glu, Leu, Pro) and “source” (Gly, Phe) AAs, ∆^15^N_bird‐fish_ are very low and around 0 except for Gly and Pro, and there is no obvious difference between those in “trophic” and “source” ones (Student`s *t*‐test, *p *=* *0.16). For essential (Ile, Leu, Phe, Val) and nonessential (Ala, Asp, Glu, Gly, Pro) AAs, there is also no obvious difference between them in ∆^13^C_bird‐fish_ (Student`s *t*‐test, *p *=* *0.08), ∆^13^C_bird‐fish_ for most AAs are around 2‰ but are ~0 if the ^13^C Suess effect (−1.8‰, Jia et al., 2013) was excluded (Figure 5) as bird bones are historical samples but fish bones are in the present. However, we need to point out that what seabirds consume is fish muscle but we used fish bone collagen samples, so we did not calculate the TDFs as they would be meaningless in this analysis.

### 3.4 TP of seabirds in different periods

**Figure 6:**
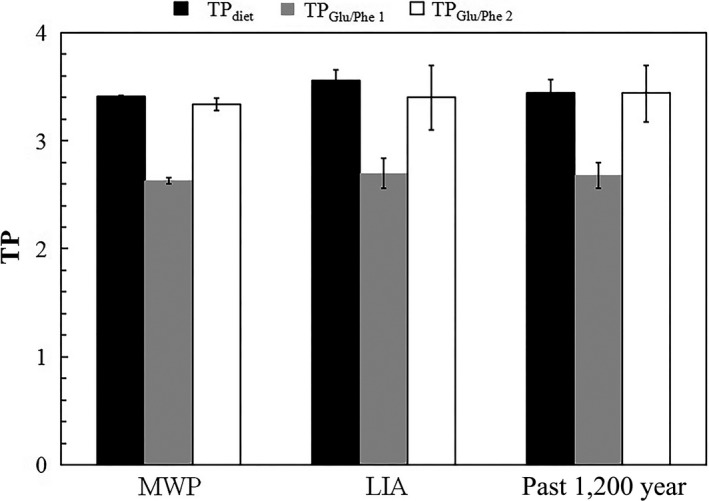
Trophic position (TP) of ancient tropical seabirds calculated from the composition of their diet (formula (1), TP_diet_), formula (3) (TP_G__lu/Phe 1_), and formula (5) (TP_G__lu/Phe 2_), in different periods [Medieval Warm Period (MWP); Little Ice Age (LIA)]

**Figure 7:**
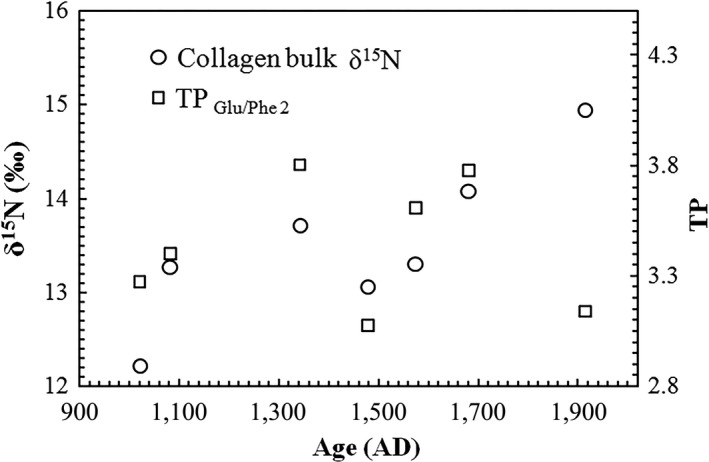
Collagen bulk δ^15^N value and the trophic position (TP) calculated from formula (5) for ancient seabird bone samples

The TP of seabirds during the MWP, LIA, and the past 1,200 years calculated from formulas (1), (3), and (5) are shown in Table 3 and Figure 6. Bone collagen δ^15^N values and the TP calculated from formula (5) (TP_Glu/Phe 2_) versus age are also plotted in Figure 7. The TP of seabirds from formula (5) (TP_Glu/Phe 2_) overall changed consistently with bone bulk δ^15^N values.

**Table 3:** Trophic position (TP) of ancient tropical seabirds calculated from the composition of their diet [formula (1), TP_diet_], formula (3) (TP_Glu/Phe 1_), and formula (5) (TP_Glu/Phe 2_). The composition of the seabird diet is taken from Wu, Liu, Fu, Xu, Li, et al. (2017)

Period	Seabird diet	TP_diet_	TP_Glu/Phe 1_	TP_Glu/Phe 2_
MWP	Flying fish: 88 ± 2%; squid: 12%	3.41 ± 0.01	2.63 ± 0.03	3.34 ± 0.06
LIA	Flying fish: 37 ± 30%; squid: 63%	3.56 ± 0.10	2.70 ± 0.14	3.40 ± 0.30
Past 1,200 years	Flying fish: 80 ± 40%; squid: 20%	3.44 ± 0.13	2.68 ± 0.12	3.44 ± 0.26

## 4 DISCUSSION

### 4.1 Nitrogen and carbon trophic enrichment in AAs

We first analyze the TEFs in AAs between bird and fish bones to preliminarily discuss the tissue‐specific isotopes including AAs isotopes for seabirds and fish. The TEF values (Figures 4 and 5) between tropical seabird and flying fish bone AAs δ^15^N or δ^13^C values were quite different from those reported in previous studies of birds (Hebert et al., 2016; McMahon et al., 2015). For example, the avian‐specific nitrogen TEF of Glu was higher (TEF_Glu_ = 3.8 ± 0.6‰) in previous study (McMahon et al., 2015). We initially attributed this to the different organs and tissues used among the different studies. Many studies have identified bulk δ^15^N and δ^13^C discrepancies (isotopic fractionation) among different tissue constituents, including avian and fish tissues (e.g., Cano‐Rocabayera et al., 2015; Hobson, 1995; Thorrold, Campana, Jones, & Swart, 1997; Wu, Xu, et al., 2017). The average flying fish bulk collagen δ^15^N value in the present study was 7.3 ± 0.5‰, which was confusing because the average seabird bone collagen bulk δ^15^N was 13.5 ± 0.8‰ and should be at a higher trophic level than flying fish, although the actual bulk δ^15^N difference (13.5‰–7.3‰ = 6.2‰) was very large. However, considering that the average flying fish muscle bulk δ^15^N value was 10.1 ± 0.2‰, it was reasonable. Thus, the method used to estimate TP based on a compound‐specific ^15^N analysis of AAs has merit in this respect, and we obtained a reasonable result from all muscle, bone, or other tissue samples of the organisms investigated (Hoen et al., 2014; Nielsen et al., 2015; and this study). As seabird food source is mainly flying fish muscle rather than fish bone collagen, we did not use the TDF value of our data, but cite the result from another study to estimate seabird TP.

In our data, there were larger TEF values for δ^13^C than reported in a previous study, in which δ^13^C TEF values between Gentoo penguins and their food source, Atlantic herring (*Clupea harengus*), were around 0 for essential AAs (Ile, Val, Phe, Leu, etc.), and were also very small (0‰–2.4‰) for nonessential AAs (Gly, Ser, Ala, Asp, Glu, Pro, etc.) (McMahon et al., 2015). The δ^13^C TEF values in the present study were higher for all essential and nonessential AAs, except Ala and Gly, and the TEF for bulk δ^13^C (2.6 ± 1.3‰) was higher than reported previously (1.0 ± 0.3‰) (McMahon et al., 2015). In addition to isotopic fractionation among different tissue samples, we suggest that the ^13^C Suess effect caused by fossil fuel combustion and carbon emissions with fewer ^13^C isotopes, also led to this difference and is about −1.8‰ (modern‐day data in 2014 minus the data for the period before 1850 AD) in the northwestern South China Sea based on a widely used model (Jia et al., 2013). Almost all of our seabird bone samples were from the period before 1850 AD, but the flying fish were collected in 2014. The collagen AAs δ^13^C differences between ancient seabird and modern‐day flying fish was first attributed to the ^13^C effect, and the differences were not due to the AAs being essential or nonessential in our study. There was a possible exception for Ala and Gly because, first seabirds predominantly consume fish muscle, whereas we used bone collagen in the study, and second, squid has accounted for 20% of the diet of the tropical seabirds over the past 1,200 years (Wu, Liu, Fu, Xu, Li, et al., 2017). Because the δ^13^C value changed little from prey to predator (DeNiro & Epstein, 1978), the average TEF value for δ^13^C of less than 1‰ (when the Suess effect was excluded) also demonstrated, once again, that the birds in our samples were likely to mainly feed on flying fish (or at least marine organisms) and were tropical seabirds. Terrestrial plants could not contribute to these food sources because plants in the Xisha Islands have an average δ^13^C value of −28.93 ± 0.81‰ (Wu, Liu, & Xu, 2017), while for our bird bone bulk sample it was −13.3 ± 0.8‰, and for compound‐specific AAs it ranged from −24.6‰ to −8.8‰.

### 4.2 Comparisons for TP calculations

Flying fish prey on phytoplankton and zooplankton (Wu, Xu, et al., 2017), which have a TP of 1 and ≥2 (~2.5 in some studies), respectively (Rybczynski, Walters, Fritz, & Johnson, 2008; Sommer et al., 2005). Thus, the TP of flying fish should be between 2 and 3 and our result (2.38 ± 0.10) is therefore quite reasonable (Table 1). Other studies also proved the rationality of this result, for example, Choy, Popp, Hannides, and Drazen (2015) reported that *Exocoetus volitans* had a ∆δ^15^N_Glu‐Phe_ of 17.4‰, which is quite consistent with that in our study (15.9 ± 0.9‰), Mancini and Bugoni (2014) summarized that δ^15^N in flying fish is ~3‰ higher than that in plankton and the bulk isotope ratio of nitrogen discriminates at 2–5‰ in each TP. The results of a nitrogen isotope mass balance indicate that flying fish feed primarily on phytoplankton (at least 62 ± 10%) and secondly on zooplankton (at most 38 ± 10%) (Saito, Johnson, Bartholow, & Hanna, 2001; Wu, Liu, Fu, Xu, Li, et al., 2017). The derived TP (2.67 ± 0.10) of squid (*L. chinensis*) is similar to that of cuttlefish *Spirula spirula* at 2.5–2.8 (Ohkouchi et al., 2013). Our results generally corresponded to the TPs of marine organisms with similar food sources, which can be inferred from a traditional stable isotope or diet analysis (Lin, 2013). Thus, formula (3) and the single TDF_Glu‐Phe_ approach were applicable for the aquatic organisms investigated in our study.

When we calculated the TP of tropical seabirds based on a compound‐specific δ^15^N analysis of AAs, we found that the results using formula (3) were unreasonable, with the average value of 2.68 ± 0.12 being only slightly higher than that of flying fish (2.38 ± 0.10). Therefore, the conventional literature value (7.6‰) and widely used formula (3) are not applicable to avian species, although they work well for aquatic organisms (e.g., Bradley et al., 2015; Nielsen et al., 2015; Ohkouchi et al., 2013). However, the average of 3.44 ± 0.26 obtained using formula (5) was nearly the same as that based on the composition of the diet (3.44 ± 0.13) (Tables 2 and 3, Figure 6). Thus, we suggest that the multi‐TDF_Glu‐Phe_ approach and formula (5) from McMahon et al. (2015) are applicable for tropical seabirds. The avian‐specific nitrogen TDF of Glu and Phe (TDF_Glu‐Phe_ = 3.5 ± 0.4‰) was significantly lower than the conventional value reported in the literature (7.6‰), because of the relatively low TEF of the trophic AA Glu (McMahon et al., 2015). According to previous studies, there are several possible reasons for the lower TDF_Glu‐Phe_ values of birds than other taxa, for example, birds grow rapidly and trophic AAs could be less enriched in growing animals; birds with a high TP have high quality food sources (rich in protein and similar AA compositions with birds), and nitrogen is excreted by birds through the production of ^15^N‐enriched urea and uric acid (Germain et al., 2013; McMahon & McCarthy, 2016). Although formula (5) was derived from penguins, it is suitable for use with tropical seabirds in the South China Sea. The two groups of birds are quite similar in some aspects; for example, penguins and tropical seabirds prey on marine fish and squid. This similar feeding habit may account for the similar nitrogen TDFs (TDF) of AAs. Thus, we can estimate the actual TP of tropical seabirds based only on the δ^15^N values in their tissue Glu and Phe, which is a simpler and more convenient method. Although the very small nitrogen isotope difference between TEF‐Glu and TEF‐Phe for flying fish and tropical seabirds bones (Figure 4), not 3.5‰ as revealed by penguin feathers, and implies that AAs δ^15^N in organisms is tissue specific; this did not have an impact on the use of formula (5) to estimate TP.

### 4.3 TP of tropical seabirds in the past

The TP for each seabird bone sample was calculated based on formula (5) (Table 2). In our previous study, seabirds from the MWP and LIA were combined to compare their relative TP (Wu, Liu, Fu, Xu, Li, et al., 2017). In this manner, the size of the seabirds, which would affect the TP (e.g., Olsson et al., 2000), was excluded. The results based on an analysis of their diet suggested that tropical seabirds were at a TP of 3.41 ± 0.01 in the MWP, and 3.56 ± 0.10 in the LIA. Similarly, the calculations based on formula (5) indicated that seabirds were at a TP of 3.34 ± 0.06 and 3.40 ± 0.30 in the MWP and LIA, respectively. The difference from Wu, Liu, Fu, Xu, Li, et al. (2017) was attributed to a change in their diet, with seabirds preying more on squid, which is at a higher TP, than flying fish in the LIA, while they mainly fed on flying fish in the MWP and in the present‐day (Wu, Liu, Fu, Xu, Li, et al., 2017). This change in their diet was a result of changes in population size, with fewer seabirds in the MWP and the flying fish population therefore being sufficient to feed them. However, there was a larger seabird population size in the LIA (Wu, Liu, Fu, Xu, Li, et al., 2017; Xu et al., 2016), at a time when flying fish were not as abundant and more squid was consumed in the diet of seabirds.

From Figure 7, the TP of seabirds from formula (5) (TP_Glu/Phe 2_) changed consistently with bone bulk δ^15^N values, except for the most recent (AD 1913) sample, which was probably more affected by human disturbance, for example, the presence of people on the islands. The similar trends suggest that both bulk δ^15^N and the multi‐TDF_Glu‐Phe_ approach have the potential to reflect the TP of seabirds. However, the bulk δ^15^N values only reflect the relative TP and background δ^15^N changes must also be known. Fortunately, the multi‐TDF_Glu‐Phe_ approach (TP_Glu/Phe 2_) provides a quantitative TP with no additional conditions, and we can also distinguish the changes in background δ^15^N values and TP variances of seabirds from δ^15^N_Glu_ and δ^15^N_Phe_. Because seabirds are widely distributed in the tropics and play a key role in the evolution of coral island ecosystems, our study is relevant to many other regions and can be used to inform other studies of the stable isotope ecology of tropical seabirds and coral island ecosystems.

## CONFLICT OF INTEREST

The authors have no conflict of interests to declare.

## AUTHOR CONTRIBUTIONS

Libin Wu, Xiaodong Liu, and Liqiang Xu designed the study and prepared the manuscript. Xiaodong Liu, Libin Wu, and Liqiang Xu collected the samples. Libin Wu, Linjie Li, and Pingqing Fu performed the experiments. All authors contributed to discussion of the results.

## DATA ACCESSIBILITY

All data used in this manuscript are present in the manuscript.
